# THE ROLE OF PSYCHOLOGICAL AND BEHAVIORAL FACTORS IN FEELING WELL-PREPARED FOR DEATH AND DYING

**DOI:** 10.1093/geroni/igae098.0374

**Published:** 2024-12-31

**Authors:** Fabio Selovin, Fiona Rupprecht, Jana Nikitin

**Affiliations:** Institute of Psychology / Friedrich Schiller University Jena, Jena, Thuringen, Germany; University of Vienna, Vienna, Wien, Austria; University of Vienna, Vienna, Wien, Austria

## Abstract

In previous research, death preparation has usually been defined as the amount and type of formal death arrangements an individual has undertaken (e.g., having written a last will). In contrast to such formal preparation, our study focuses on the subjective feeling of being prepared for death and dying. Based on construal-level theory, we assume that different behavioral and psychological factors matter for this subjective feeling of preparedness depending on the subjective temporal distance to death. The sample consisted of 121 participants aged 51 to 95 years (M = 63, SD = 9; 48% female). Participants were asked how prepared they felt for death and dying and how extensive they experienced their remaining future. Furthermore, we assessed aspects of formal death preparation, informal death preparation (i.e., death communication with partner), generativity and death attitudes (i.e., fear of death, belief in an afterlife). Results showed that neither formal nor informal death preparation were predictive of the subjective feeling of being prepared. Instead, the fear of death emerged as the strongest (negative) predictor across the sample. Interaction analyses further showed that individuals who felt close to death, felt prepared when their generativity and opportunities for being generative were high. Findings are discussed using a life-span perspective, illuminating what being prepared for death and dying might mean for the individual.

